# Phosphorylated *α*-Synuclein-Copper Complex Formation in the Pathogenesis of Parkinson's Disease

**DOI:** 10.1155/2017/9164754

**Published:** 2017-11-23

**Authors:** Juan Antonio Castillo-Gonzalez, Maria De Jesus Loera-Arias, Odila Saucedo-Cardenas, Roberto Montes-de-Oca-Luna, Aracely Garcia-Garcia, Humberto Rodriguez-Rocha

**Affiliations:** ^1^Departamento de Histologia, Facultad de Medicina, Universidad Autonoma de Nuevo Leon, 64460 Monterrey, NL, Mexico; ^2^Centro de Investigaciones Biomedicas del Noreste, Monterrey, NL, Mexico

## Abstract

Parkinson's disease is the second most important neurodegenerative disorder worldwide. It is characterized by the presence of Lewy bodies, which are mainly composed of *α*-synuclein and ubiquitin-bound proteins. Both the ubiquitin proteasome system (UPS) and autophagy-lysosomal pathway (ALS) are altered in Parkinson's disease, leading to aggregation of proteins, particularly *α*-synuclein. Interestingly, it has been observed that copper promotes the protein aggregation process. Additionally, phosphorylation of *α*-synuclein along with copper also affects the protein aggregation process. The interrelation among *α*-synuclein phosphorylation and its capability to interact with copper, with the subsequent disruption of the protein degradation systems in the neurodegenerative process of Parkinson's disease, will be analyzed in detail in this review.

## 1. Introduction

Parkinson's disease (PD) is the second most frequent neurodegenerative disorder related to aging worldwide [1]. The clinical symptoms of this disease are resting tremor, rigidity, bradykinesia, akinesia, postural instability, difficulty in speech, and breathing problems [2]. Most PD cases appear to be sporadic, and only about 5–10% of the cases are due to genetic mutations [3]. Exposure to environmental pollutants such as herbicides (paraquat), pesticides (rotenone), and toxic substances during the manufacture of narcotic drugs (MPTP^+^) and prolonged exposure to transition metals have been reported to be related to sporadic cases of PD [4–6]. PD is characterized by dopaminergic neuronal loss in the substantia nigra at the central nervous system (CNS), a significant reduction in dopamine levels, and the presence of Lewy bodies [7, 8]. Lewy bodies are composed of abnormal deposits of protein aggregates, particularly *α*-synuclein and ubiquitin-bound proteins [9]. Abnormal protein aggregation results from the UPS and ALS alteration, and the latter includes disruption of lysosomal hydrolase trafficking [10–12]. Interestingly, some metal ions such as copper have shown to promote the protein aggregation process [13–15]. Additionally, phosphorylation of *α*-synuclein, along with copper, accelerates the protein aggregation process [16, 17].

Therefore, in order to have a better understanding of the mechanisms involved in the neurodegenerative process of PD, the interrelation among *α*-synuclein phosphorylation and its capability to interact with copper, as well as the consequent disruption of the protein degradation systems, will be analyzed in detail in this review.

## 2. *α*-Synuclein

**Figure 1:**
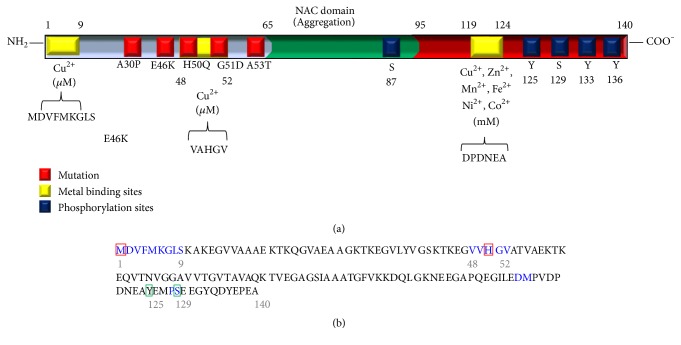
*Schematic structure of α-synuclein*. (a) *α*-Synuclein mutations related to familial PD are shown as red squares. Metal-binding sites are depicted as yellow squares. Seine (S) and threonine (Y) amino acid residues targeted by phosphorylation are indicated as blue squares. (b) Amino acid composition of *α*-synuclein. Residues in blue represent copper-binding sites. Red squares indicate methionine 1 and histidine 50, which are independent anchoring sites for copper binding. Green squares show phosphorylation sites (Y125 and S129) related to an increased copper-binding capability.

The main histological hallmark of PD is the presence of eosinophilic cytoplasmic inclusions known as Lewy bodies, which are localized in the substantia nigra and are formed mostly by *α*-synuclein [18–20]. *α*-Synuclein is a thermostable, preserved, and unfolded cytosolic protein [21], belonging to a family of homologous proteins called synucleins, and is expressed in approximately 80% of the total area of the human brain [22, 23]. *α*-Synuclein consists of 140 amino acid residues [24] organized in three structural regions: an amphipathic amino-terminal domain from 1 to 60 amino acid residues, responsible for the binding of *α*-synuclein to lipid vesicles [25, 26]; the NAC (non-amyloid-*β* component) region from 61 to 95 amino acid residues, also found in amyloid plaques of patients suffering from Alzheimer's disease [27] and responsible for *α*-synuclein aggregation and *β* sheets arrangement [28]; and the carboxy-terminal domain from 96 to 140 amino acid residues, which is the main target for the protein phosphorylation [29, 30] (Figure 1(a)). *α*-Synuclein is primarily expressed in neurons at cytosolic level and is abundant in presynaptic terminals [31]. However, it has also been linked to synaptic vesicles, plasma membrane lipid rafts and the nucleus [32]. Up to date, the main normal function of *α*-synuclein has not been well defined. *α*-Synuclein has been related to different functions, including inhibition of tyrosine hydroxylase [33], inhibition of dopamine release [34], dopamine uptake [35], neural plasticity, synaptic maturation and maintenance [36–38], and v-SNARE complex assembly [39, 40].

*α*-Synuclein has the capability to assemble into amyloid fibers, soluble oligomers, and/or aggregates. Once the accumulation of *α*-synuclein surpasses its degradation rate, it leads to the formation of Lewy bodies and the subsequent death of dopaminergic neurons in the substantia nigra [41]. It has been suggested that *α*-synuclein protofibrils are responsible for the neurotoxic effects induced by *α*-synuclein [42, 43]. Among the possible mechanisms involved in the neurotoxicity mediated by *α*-synuclein are the following: mitochondrial dysfunction, oxidative stress [44], lysosomal leakage [45], cytoskeletal disruption [46], altered axonal transport, and subsequent synapses dysfunction, which are all related to neurodegeneration [47]. *α*-Synuclein is targeted for degradation by the UPS and the ALS including the chaperone-mediated autophagy (CMA) and macroautophagy (autophagy) [48–51]. Importantly, both degradation pathways are dysregulated or inhibited in PD [52].

The relationship between *α*-synuclein and PD was established with the identification of specific mutations in the SNCA gene encoding for *α*-synuclein, in families with PD. The specific mutations identified in *α*-synuclein were a substitution from alanine to threonine at amino acid residue 53 (A53T), a mutation of Greek origin [53]; a substitution from alanine to proline at amino acid residue 30 (A30P), a mutation of Germanic origin [54]; and a substitution from glutamic acid to lysine at amino acid residue 46 (E46K), a mutation of Spanish origin [55] (Figure 1(a)). Recently, two new mutations have been identified, a substitution from histidine to glutamine at amino acid residue 50 (H50Q) [56] and a substitution from glycine to aspartic acid at amino acid residue 51 (G51D), a mutation of French origin [57]. Additionally, SNCA gene duplication [58] and triplication also occur and are related to PD [59].

## 3. *α*-Synuclein Phosphorylation in Parkinson's Disease: Neuroprotective or Neurotoxic?

In the aggregation process of *α*-synuclein, its phosphorylation plays an important role [29, 60] by directing its localization and interaction [61] and by modifying its secondary and tertiary conformation [62–65]. *α*-Synuclein is targeted by phosphorylation on multiple sites located at its carboxy-terminal end (S87, S129, Y125, Y133, and Y136) [66–71] (Figure 1). Several kinases have been linked to *α*-synuclein phosphorylation, such as casein kinases 1 and 2 (CK1 and CK2), G protein-coupled receptor kinases 2 and 5 (GRK2 and GRK5), polo-like kinase 2 (PLK2) [29, 72, 73], Fyn [74], and more recently serine/threonine protein kinase (LK6) and MAP kinase-interacting kinase 2a (Mnk2a) [75].

**Figure 2:**
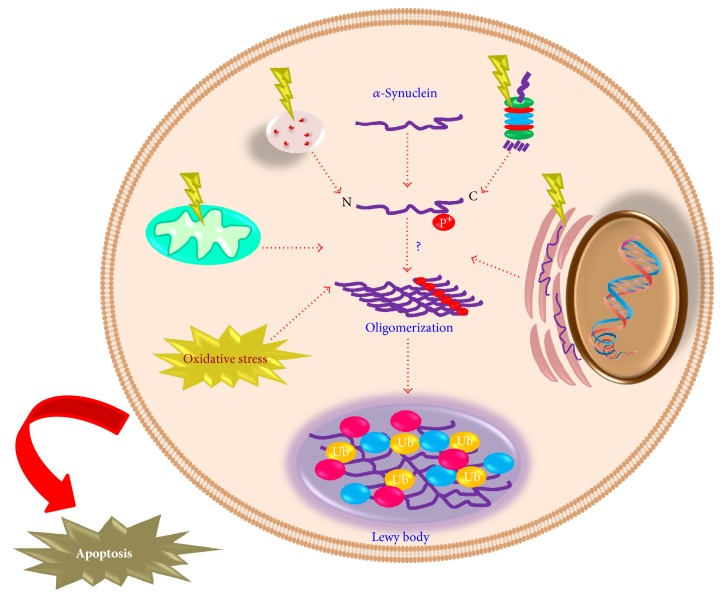
*Cell alterations involved in the aggregation process of α-synuclein*. Damaged or unrequired proteins are regulated by both the proteasomal and lysosomal degradation pathways. UPS disruption leads to activation of the ALS and vice versa, as a compensation mechanism. Both mechanisms are affected in PD, which results in protein accumulation including *α*-synuclein and ubiquitin-bound proteins. Accumulation of unfolded or misfolded proteins into the endoplasmic reticulum activates the unfolded protein response. Mitochondrial dysfunction and oxidative stress are also interrelated and linked to the pathogenesis of PD. All these alterations are associated with the phosphorylation process of *α*-synuclein and increase *α*-synuclein oligomerization, leading to Lewy body formation and subsequent apoptotic cell death.

Studies performed in cell cultures with neuronal phenotype have demonstrated that CK2-mediated *α*-synuclein phosphorylation, particularly at S129, increases the appearance of eosinophilic cytoplasmic inclusions resembling the Lewy bodies of PD [76]. A major component of these inclusions consists of C-terminally truncated *α*-synuclein, and lysosomal proteases, such as cathepsin D, may be involved in its production for *α*-synuclein oligomerization [77]. Some mechanisms are triggered by the phosphorylation of *α*-synuclein, including the unfolded protein response (UPR) and disruption of lysosomal degradation pathways, which may lead to protein aggregation and subsequently to cell death (Figure 2) [78, 79]. Monomers and dimers of *α*-synuclein are degraded by ALS, specifically, CMA [79, 80]. In addition, it has been reported that a phosphorylated-like mutant version of *α*-synuclein (S129E), which mimics the biochemical and biophysical properties of *α*-syn phosphorylation observed in PD patients' brains [76] and remained “phosphorylated-like” after exposure to the lysosomal fraction, cannot translocate across the lysosomal membrane probably because of a conformational change induced by its phosphorylation, decreasing its interaction with the CMA receptor (LAMP-2A) at the lysosomal membrane [79, 80].

**Figure 3:**
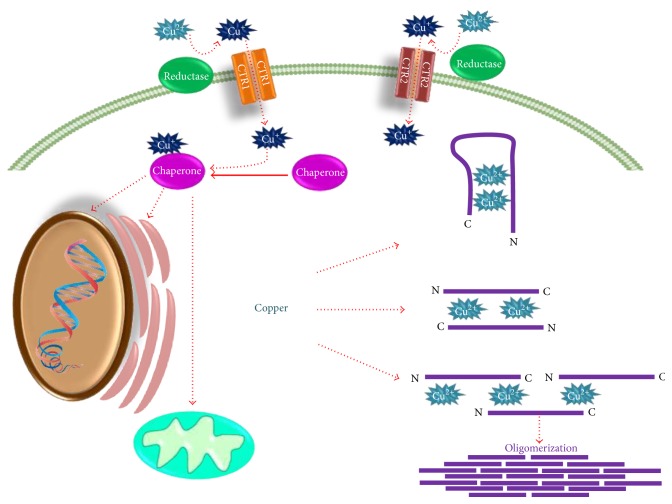
*α-Synuclein-copper complex formation process*. Copper can be found in living organisms in both forms, oxidized Cu^2+^ and reduced Cu^+^, and enters into the cell as Cu^+^ through CTR1 and CTR2. Afterwards, copper is transported to the nuclei, endoplasmic reticulum, and mitochondria via chaperone proteins. An overload of copper may lead to the *α*-synuclein-copper complex formation by three potential mechanisms. In the first one, a single *α*-synuclein molecule binds to Cu^2+^, folding and bringing together the amino and carboxy-terminal ends. The second mechanism involves two molecules of *α*-synuclein with a head-to-tail arrangement, generating a copper-binding site at both ends. In the third mechanism, the carboxy-terminal region of one molecule of *α*-synuclein interacts with the amino-terminal region from another molecule of *α*-synuclein creating a Cu^2+^ binding site. Next, one of the two *α*-synucleins interacts with a third *α*-synuclein molecule, forming a second Cu^2+^ binding site. This process will eventually lead to *α*-synuclein oligomerization.

In addition, dysfunctional mitochondrial metabolism and increased ROS production are also related to the phosphorylation of *α*-synuclein (Figure 3) [81]. Hydrogen peroxide- (H_2_O_2_-) induced oxidative stress increases the phosphorylation of *α*-synuclein at S129 and the formation of cytoplasmic inclusions [76]. On the other hand, some neurotoxins and the UPS inhibition increase the activity of GRK5 and CK2, whose interaction with Ca^2+^/calmodulin increases *α*-synuclein phosphorylation at S129 [82–84]. Rotenone, an inhibitor of mitochondrial complex I, along with iron, increases the levels of *α*-synuclein phosphorylation at S129, by inducing ROS production in dopaminergic cells [81].

So far, the role of *α*-synuclein phosphorylation is controversial. Some studies have shown a neuroprotective role of *α*-synuclein phosphorylation at S129 by preventing the binding of *α*-synuclein oligomers to membranes and, therefore, cellular disruption [85–88]. Additionally, phosphorylation at S129 blocked *α*-synuclein fibrillation* in vitro* [89]. Many studies had focused on the role of *α*-synuclein phosphorylation, specifically at S129, and also at other residues such as S87. *α*-Synuclein mutant variants, capable of mimicking or inhibiting the phosphorylation process (S129D, S129E, and S129A), have contributed to the elucidation of its role [66, 70, 89–92]. Phosphomimic mutants S129D/E were not able to reproduce* in vitro* the structural and aggregation properties of *α*-synuclein. However, a nonphosphomimic mutant S129A showed a higher protein aggregation rate and neurotoxicity than the wild type form [70, 71, 89].

On the contrary, there is evidence showing that *α*-synuclein phosphorylation at S129 induces cytotoxicity [66, 77, 93, 94]. It has been demonstrated that *α*-synuclein phosphorylation at S129 mediated by CK2 is an important factor for its protein aggregation and toxicity, inducing UPR dysregulation, endoplasmic reticulum (ER) stress, and apoptosis [78]. Besides, phosphorylation at S129 is essential for interaction of *α*-synuclein with synphilin-1 and parkin, which form the ubiquitinated inclusions [76]. Recently, it has been reported that *α*-synuclein can also be phosphorylated by LK6 and Mnk2a, with subsequent dopaminergic neuronal death and formation of cytoplasmic inclusions, respectively [75]. Nonetheless, it has been suggested that malfunction of the UPS increases CK2 activity, resulting in hyperphosphorylation of the *α*-synuclein at S129 [95].

Approximately 90% of *α*-synuclein detected in Lewy bodies from postmortem PD samples is phosphorylated at S129. Conversely, only 4% of *α*-synuclein present in normal brains is phosphorylated [66, 70, 96, 97]. Importantly, a mass spectrometry study in human cerebrospinal fluid (CSF) of PD and other parkinsonian disorders determined a significantly higher concentration of phosphorylated *α*-synuclein at S129, as well as a significant increase in the ratio of phosphorylated *α*-synuclein at S129/total *α*-synuclein in PD compared to healthy controls [98]. More recently, a marked difference between PD patients and healthy controls was observed with a sensitive and specific Elisa test, by combining measurements of total, oligomeric, and phosphorylated (S129) *α*-synuclein in CSF [99].

## 4. Interaction of Phosphorylated *α*-Synuclein with Metal Ions

Proteins are the main biomolecules affected in most pathologies; posttranslational modifications of proteins suchlike oxidation, nitration, carbonylation, glutathionylation, and phosphorylation are related to protein inactivation. Phosphorylated proteins have a strong binding affinity to certain metals [16, 100, 101]. Multivalent metal ions, like manganese, cobalt, iron, and mainly aluminum and copper, increase *α*-synuclein fibril formation by inducing conformational changes [14, 102, 103]. *α*-Synuclein may interact with different metal ions at either its carboxy-terminal domain or its amino-terminal domain, depending on the metal ion concentration [13]. For instance, at low concentrations (40–100 *μ*M) Cu^2+^ ion binds to the amino-terminal domain [13, 104], while at extremely high concentrations (0.5–5 mM), which are unlikely to occur in tissues, metal ions such as Fe^2+^, Mn^2+^, Ni^2+^, Co^2+^, and Cu^2+^ bind to the carboxy-terminal domain [105]. Cu^2+^ ion is a potent inducer and accelerator of *α*-synuclein aggregation, linked to the carboxy-terminal domain, which is required for its oligomerization [106]. Phosphorylation at both Y125 and S129 residues of *α*-synuclein, which are close to metal-binding sites, increments Cu^2+^, Pb^2+^, and Fe^2+^ binding capability to carboxy-terminal domain (Figure 1) [17, 105].

## 5. Copper Mediates *α*-Synuclein Aggregation

On the other hand, copper has the ability to inhibit the proteasomal chymotrypsin-like peptidase activity [107]. Copper enters into the cell through the copper transporters 1 and 2 (CTR1 and CTR2), which are located on the cell membrane (Figure 3) [108]. Two regions, ^1^MDVFMKGLS^9^ and ^48^VVHGV^52^ (Figure 1), with high-affinity binding sites for copper were identified at *α*-synuclein, and may be of great biological importance in the pathogenesis of PD [104]. Within the *α*-synuclein sequence, methionine 1 and histidine 50 residues function as independent anchoring sites for copper binding (Figure 1) [104, 109, 110].

There are three models that have been suggested for copper binding to *α*-synuclein (Figure 3). In the first model, a single *α*-synuclein molecule binds to Cu^2+^, folding and bringing together the amino and carboxy-terminal regions. The second model involves two molecules of *α*-synuclein with a head-to-tail arrangement, generating a copper-binding site at both ends. In the third model, *α*-synuclein oligomerization takes place by interaction of the carboxy-terminal region of one molecule of *α*-synuclein with the amino-terminal region from a second molecule of *α*-synuclein originating a Cu^2+^ binding site; then a second Cu^2+^ binding site is formed by interaction of one of the two *α*-synucleins with a third *α*-synuclein molecule [15].

Regarding the aggregation process of *α*-synuclein mediated by copper, two mechanisms have been proposed. In one of them, high levels of *α*-synuclein-copper complexes will cause instability of intramolecular interactions leading to self-assembling of *α*-synuclein into fibrillar complexes. In the second one, copper redox-mediated reactions induce oxidation of *α*-synuclein using electron donors (NADH, NADPH, glutathione, etc.), causing its oligomerization and precipitation [111–114].

Environmental exposure to metal ions (e.g., zinc and copper) induces *α*-synuclein aggregates and oxidative stress, which are also associated with dysregulation of the UPS in PD [82, 115, 116].

Copper plays a dual role in the neurotoxic effect of *α*-synuclein. Once intracellular copper concentration is raised, chaperone proteins (e.g., ATOX1, CCS, MT3, and COX 17) are in charge to uptake this metal inside the cell, but an overload of copper might surpass the chaperone proteins available to regulate its levels. On the other hand, mutations affecting the ability of chaperones to bind copper might also increase its toxic effect [117]. Subsequently, free copper binds to the UPS to inhibit its activity; then *α*-synuclein is phosphorylated increasing its affinity to metals [71]. *α*-Synuclein-copper complex formation alters cell redox signaling, which results in ROS formation including H_2_O_2_. H_2_O_2_ oxidizes dopamine, which is toxic to dopaminergic neurons [118, 119].

## 6. Concluding Remarks

*α*-Synuclein is a highly relevant protein in PD etiopathology, and since the elucidation of *α*-synuclein-copper interactions, this transition metal was brought into the spotlight of neurodegeneration research. Although this complex formation is now subject of intense research, many open questions remain: How are levels of copper regulated by *α*-synuclein? Does copper influence *α*-synuclein phosphorylation and aggregation? How important is copper and *α*-synuclein interaction? Can we use phosphorylation of *α*-synuclein as a biomarker? Can we exploit the inhibition of phosphorylation of *α*-synuclein as a therapeutic approach? It is certain, that these therapies need to initiate promptly in order to address pathological changes in a less advanced stage. Regrettably, the diagnosis of PD nowadays is based on purely clinical signs, and these signs are manifested when more than half of dopaminergic neurons have died. Therefore, identification of early biomarkers such as *α*-synuclein phosphorylation may be a promising approach for diagnosis and subsequently for PD treatment and correlated with preclinical signs indicating incipient disease at a nonsymptomatic stage. Since *α*-synuclein and copper play such important roles in the aggregation process, a chelator administration is currently under investigation and may be a helpful approach against PD.
